# Linking Quality of Work in Midlife to Volunteering During Retirement: a European Study

**DOI:** 10.1007/s12062-015-9129-8

**Published:** 2015-07-08

**Authors:** Morten Wahrendorf, David Blane, Katey Matthews, Johannes Siegrist

**Affiliations:** Centre for Health and Society, Institute for Medical Sociology, University of Düsseldorf, Universitätsstrasse 1, 40225 Düsseldorf, Germany; International Centre for Life Course Studies in Society and Health, Department of Epidemiology and Public Health, University College London, London, UK; Cathie Marsh Institute for Social Research, University of Manchester, Manchester, UK; Senior Professorship on Work Stress Research, Faculty of Medicine, University of Düsseldorf, Düsseldorf, Germany

**Keywords:** Voluntary work, Work stress, SHARE

## Abstract

There is increasing recognition that disadvantaged work and employment conditions over the life course, including psychosocial stress at work, have negative long-term effects on health at older ages. Yet, the question whether stress at work additionally influences the likelihood of participating in voluntary work during retirement still needs to be explored. This paper studies long-term influences of stressful work during adulthood (as defined by low control and low reward at work) on participation in voluntary work in older ages. Analyses are based on the Survey of Health Ageing and Retirement in Europe (SHARE), with detailed retrospective information on individual life courses collected among 11,751 retired men and women in 13 European countries. Results of multivariable analyses demonstrate that people who experienced stressful work are also less likely to participate in voluntary work during retirement. Importantly, these associations remain significant after controlling for important factors, including disability in older ages and disadvantaged socioeconomic circumstances. In conclusion, findings suggest that promoting good working conditions may not only increase health and well-being, but also encourage participation in productive activities after labour market exit.

**Electronic supplementary material:**

The online version of this article (doi:10.1007/s12062-015-9129-8) contains supplementary material, which is available to authorized users.

## Introduction

As a result of demographic ageing in combination with significant improvements of population health a majority of people in high income countries are now reaching their ‘third age’ without severe physical or mental impairment (Laslett 1996; Oeppen and Vaupel 2002). These older men and women are ready to continue an active life characterized by engagement in productive or leisure activities and individual freedom (Laslett 1996).

At the same time, this phase of the life course often lacks a clear societal definition in terms of social roles and social status, legitimized expectations, norms and values (Riley et al. 1994). While most middle-aged people experience a secure sense of social identity by maintaining core social roles (such as the work role, family roles or civic roles), social identity during third age, and in particular once people have retired, becomes more fragmented and insecure, often in combination with a reduced intensity of contacts in social networks. Yet, there may be options available to continue engagement in some type of productive activity during retirement (Loh and Kendig 2013), and thereby to maintain a sense of social identity and personal fulfilment. Participating in voluntary work is one such option, but it is questionable whether each individual has the same opportunity to participate in such activities.

Existing surveys from Europe indicate that about 10 % of men and women in their third age are engaged (at least once in a month) in some type of voluntary work across Europe (Erlinghagen and Hank 2006; Hank and Stuck 2008; Siegrist and Wahrendorf 2009; McMunn et al. 2009), with comparatively higher rates in Northern European countries together with the Netherlands and rather low rates in Eastern and Southern Europe. Robust evidence also demonstrates that regular participation in voluntary work is associated with tangible gains in prospective health and wellbeing (Bath and Deeg 2005; Mendes de Leon 2005; McMunn et al. 2009; Wahrendorf and Siegrist 2010). However, the rates of engagement in voluntary work vary substantially within countries (Loh and Kendig 2013; Erlinghagen and Hank 2006; Siegrist and Wahrendorf 2009). In particular, social gradients of participation have been consistently reported, with a lower prevalence among men and women with lower education, lower income or who work in lower-skilled occupations (Siegrist and Wahrendorf 2009; Loh and Kendig 2013). These observations point to the fact that the structure of societal opportunities as well as the motivations and capabilities of people reaching their third age determine the probability of engaging in voluntary work (and of sharing associated benefits). In other words, motivations and capabilities that are conducive to volunteering may be shaped during earlier stages of people’s life course (Tang 2006).

In this study we set out to analyse the contribution of work and employment conditions during midlife towards motivating and enabling people to be involved in volunteering during retirement. More specifically, we hypothesize that men and women who worked under favourable psychosocial work environments during midlife are more likely to engage in voluntary work after labour market exit, and that this association is mainly due to a favourable quality of work that reinforces the motivations and capabilities of pursuing productive activities during retirement. Given these assumptions, the crucial question is how we can define a favourable psychosocial work environment.

It seems essential that work – being accomplished almost daily over many years -has to satisfy specific requirements to enable the working person to meet important psychological needs of successful self-regulation, at least to a significant extent. Among these psychological needs, two are particularly important in the context of work and employment: the need of experiencing autonomy and control (Karasek and Theorell 1990), thus strengthening a favourable sense of self-efficacy (Bandura 1997), and the need of experiencing recognition and justified reward (Siegrist 1996), thus strengthening a positive sense of self-esteem (Deci and Ryan 1985). The ‘Demand-Control’ model and the ‘Effort-Reward Imbalance’ models are respective theoretical models of work stress that identify psychosocial work environments meeting or failing to meet these human needs.

The former model describes specific job task profiles that are favourable or detrimental to the health and wellbeing of working people. In the case of favourable task profiles, jobs offering a high level of decision latitude and skill discretion strengthen self-efficacy and wellbeing, even in combination with high work demands. In contrast, jobs with high demands that offer little or no control over one’s tasks and that avert skill development prevent workers from experiencing self-efficacy and wellbeing (Karasek and Theorell 1990).

The model of effort-reward imbalance builds on the notion of social reciprocity that lies at the core of the employment (or work) contract. Experiencing appropriate rewards that match efforts expended reinforces a positive sense of self-esteem and wellbeing, in conjunction with recurrently activated brain reward circuits. Conversely, violations of this balance under circumstances where efforts outmatch rewards (failed reciprocity) elicit sustained stressful experience with adverse long-term effects on health and wellbeing (Siegrist 2005).

Based on these notions we test two research hypotheses in a large data set with data on older men and women from a variety of European countries, the Survey of Health, Ageing, and Retirement in Europe (SHARE, see Methods for details).

First, we assume that men and women who experienced high control or high reward in their main job during working life have an increased probability of engaging in voluntary work during retirement, as compared to those who experienced low control or low reward during working life (hypothesis 1).

Second, we assume that the association between quality of work and volunteering remains statistically significant after adjusting for important additional factors linked to engagement in voluntary work, in particular disability and disadvantaged socioeconomic circumstances (hypothesis 2).

The first hypothesis implies that recurrent experience of autonomy and control, as well as of reward and recognition at work motivates people to engage in voluntary work, even in the absence of financial compensation, as is the case with volunteering during retirement. Implicit in the second hypothesis is the assumption that the effects of high control and high reward at work on the probability of volunteering are strong enough to persist after controlling for important influencing factors. In other words, we assume that the association between favourable psychosocial working conditions and voluntary work is not due to better physical health in older ages or to more advantage socioeconomic circumstances.

## Methods

### Data Source

We use data from the third wave (2008–09) of the Survey of Health, Ageing and Retirement in Europe (SHARE) with retrospective information on quality of work that we combine with information on volunteering in wave 2 (2006–07). SHARE is the first cross-national, longitudinal research project collecting data on a variety of sociological, economic and health-related topics among nationally representative samples of older adults in Europe. The survey started in 2004–2005 in 11 countries (Sweden, Denmark, Germany, Netherlands, Belgium, France, Switzerland, Austria, Italy, Spain, Greece), with on-going waves of data collection at 2 year intervals. Two new countries joined SHARE in wave 2 (Czech Republic and Poland).

In each country, samples consist of a probability household sample, with individuals aged 50 years or older plus their (possibly younger) partners. New cohorts (so called “refreshers”) are added subsequently to maintain population representation. At study onset the household response rate was 61.6 % for the total sample ranging from 81 % in France to 39 % in Switzerland, with rates above 50 % in 8 out of 11 countries. This is above average compared to other European Surveys (Börsch-Supan and Jürges 2005). With respect to attrition between wave 2 and wave 3, the per cent of respondents lost varied between 34 % (Austria) and 14 % (Switzerland), with rates below 20 % in seven countries (Schröder 2011).

In contrast to the first two waves, the third wave of SHARE consists of a separate retrospective survey collecting details on participants’ life course (Börsch-Supan et al. 2013), including details on previous employment histories (also called SHARELIFE). More details about SHARE and its methods are available online (www.share-project.org).

### Respondents

In total, 26.836 participants were interviewed at wave 3. Because we are interested in participation in voluntary work after working life, we restrict the sample to those who had already left the labour market when assessing participation in voluntary work. Also, respondents are only considered if they had an employment history of at least 5 years. Further, to avoid a sample bias due to selective mortality we exclude respondents older than 90 years. Finally, we exclude respondents when the interviewer documented respondent difficulties in answering the retrospective interview (about 4 % of the total sample). This results in a final sample with full available data on 5770 men and 5981 women (*N* = 11,751) born between 1916 and 1957.

### Measures

#### Stressful Work

We use two binary indicators of stressful work, one measuring low control and another low reward. In both cases respondents were asked to assess in retrospect the degree of adversity experienced in the main job of their occupational career (with a mean job length of 24.5 years in our sample). More specifically, respondents reported their level of agreement to four items (on a four-point Likert scale ranging from ‘strongly agree’ to ‘strongly disagree’), two for each domain. Items are taken from original questionnaires (Siegrist et al. 2004; Karasek et al. 1998), and are presented in a supplementary table in the appendix (table S1). When necessary, items were recoded to achieve uniform coding (higher values indicating more stress at work). To identify elevated levels of stressful work in terms of the two components, conditions are classified as stressful if respondents reported high work stress (e.g. “agree” or “strongly agree”) to both items of the respective dimension.

#### Voluntary Work

In wave 2 respondents were asked about their participation in voluntary or charity work (voluntary work). In details, respondents reported whether or not they were involved during the last 4 weeks.

#### Disability

We include two binary indicators of disability, that both were shown to be well-comparable between countries (Chan et al. 2012). The first measure of disability indicates an increased number (three or more) of reported limitations in mobility (‘Mobility limitations’), as based on a list of 10 items. These limitations include difficulties in mobility, arm functions and fine-tuned motor function. The second measure indicates 1 or more limitations in performing activities of daily living (‘ADL limitations’).

#### Socioeconomic Circumstances

We use two measures of socioeconomic circumstances, one measuring the main occupational position held during working life, in terms of skill-level, and another measuring the financial circumstances during retirement, in terms of wealth. Occupational position refers to the main job of the working career, as assessed by the ten main occupational groups of the International Standard Classification of Occupations (ISCO). For the analyses, groups were re-classified according to the four different skill-levels, representing the broad hierarchical structure of ISCO. This skill level refers to skills required in the job for a competent performance of the tasks and duties, which does not necessarily correspond to the existing educational qualification of the worker. With regard to occupational position, higher skill levels are supposed to put worker in a more advantage situation on the labour market, because jobs requiring higher skill levels are expected to be related to higher salary and more continuity of employment as compared to jobs with lower skill levels. Notably, skill levels also constitute an important aspect in more sophisticated classifications schemes, for example, to regroup employed people within the Erikson-Goldthorpe-Portocarero (EGP) class scheme (Erikson and Goldthorpe 1992).

Our measure of wealth is based on household total net worth. In addition to financial wealth (savings, net stock value, mutual funds and bonds), it also includes housing wealth (value of primary residence, other real estates and own business share and cars). For the analyses, we adjusted for household size in accordance with the OECD equivalent-scale, and categorised resulting values into country-specific tertiles (low, medium, high). Because our wealth measure includes accumulated savings and not only direct income, it may be more appropriate for older populations as an indicator of financial circumstances (all analyses were also calculated with equalized household income, and findings were similar).

#### Additional Variables

We also include age of the respondent, sex and age at retirement, which we regrouped into four categories (before 55, 55–59. 60–64, 65 or older).

### Analytical Strategy

**Fig. 1:**
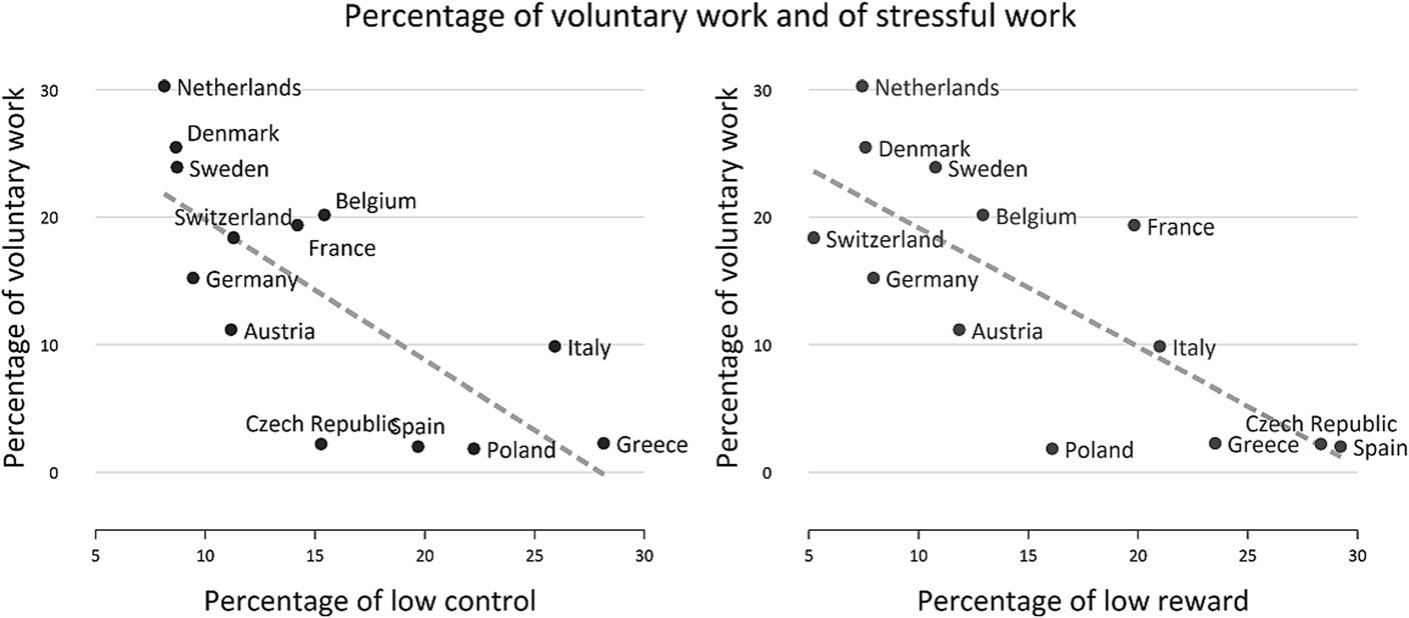
Percentage of men and women engaging in voluntary work and with poor working conditions in SHARE countries

Following sample description (Table 1), we present percentages of older people participating in voluntary work by covariates (Table 2). In addition, country-variations of participation rates are presented alongside levels of stressful work for each country (Fig. 1).

**Table 1:** Sample description: percentage and frequencies (N) or mean scores and standard deviation (SD); (N = 11,751)

Variables	Categories or range	% or (mean)	N or (SD)
Mean age	50–90	(68.31)	(8.33)
Sex	Male	49.10	5770
	Female	50.90	5981
Retirement age	Before 55	33.00	3878
	55–59	26.20	3079
	60–64	29.09	3418
	65 or older	11.71	1376
ADL-limitation	Not limited	89.67	10537
	Limited	10.33	1214
Mobility limitation	Not limited	74.83	8793
	Limited	25.17	2958
Occupational position	Very low	19.27	2264
	Low	58.06	6823
	High	9.21	1082
	Very high	13.46	1582
Wealth	Low	31.22	3669
	Medium	35.24	4141
	High	33.54	3941
Control at work	Low	16.07	1888
	High	83.93	9863
Reward at work	Low	16.27	1912
	High	83.73	9839
Voluntary work	Yes	13.74	1614
	No	86.26	10137

**Table 2:** Percentage of people participating in voluntary work by covariates (*N* = 11751)

Variables	Categories	%	Number
Sex	Male	14.49	836
	Female	13.01	778
Retirement age	Before 55	11.04	428
	55–59	13.09	403
	60–64	17.23	589
	65 or older	14.10	194
ADL-limitation	Not limited	15.07	1477
	Limited	7.02	137
Mobility limitation	Not limited	15.99	1406
	Limited	7.03	208
Occupational position	Very low	8.92	202
	Low	12.28	838
	High	18.76	203
	Very high	23.45	371
Wealth	Low	11.07	406
	Medium	12.99	538
	High	17.00	670
Control at work	Low	8.16	154
	High	14.8	1460
Reward at work	Low	7.9	151
	High	14.87	1463
Total		13.74	1614

Thereafter, to test our hypotheses, we calculate a series of multivariable logistic regression models using participation in voluntary work as the outcome variable. In sum, we estimate six different models, all adjusted for sex, country affiliation (country dummies), retirement age (four categories) and age of the respondent. In the first four models we study the role of the two indicators of stressful work - before and after adjustment for the two indicators of disability. In Model 5 associations for the two indicators of socioeconomic circumstances (again adjusted for disability) are presented and a final model includes all variables simultaneously. Given that previous research has shown that engagement in voluntary work varies non-linearly across the life course (Loh and Kendig 2013), we include two fractional polynomial (FP) transformations for age (Schmidt et al. 2013; Royston and Sauerbrei 2008) and use the “fracpoly” procedure in STATA. This has several advantages compared to categorizing age into age groups (e.g. loss of information, inflated number of parameters) and allows for a more flexible non-linear modulation of the age function as compared to conventional polynomials. More specifically, power terms are not limited to positive integers only but can also be negative and fractional (e.g. x^-1.5^). Furthermore, powers are not fixed and defined by the researcher, but selected by an automated process that compares models with different powers and identifies polynomials that best predicts voluntary work as a function of age (based on deviance statistics).

In the results, the estimated regression models are presented in Table 3. Following recommendations of Mood (2010), we not only present odds ratios (OR) and their levels of significance, but also show the average marginal effects (AME) (Williams 2012). On the one hand, these are more intuitive and easier to interpret as compared to OR (AME indicate the predicted percentage difference between two groups, for example, between men and women). On the other hand, they can be compared across different models (Mood 2010).

**Table 3:** Associations between covariates and voluntary work: results of logistic regression models: Odds ratios (OR), confidence intervals (CI) and average marginal effects (AME) (*N* = 11,751)

Variables	Categories	Model 1			Model 2			Model 3			Model 4			Model 5			Final model		
		OR	(CI 95 %)	AME	OR	CI 95 %	AME	OR	CI 95 %	AME	OR	CI 95 %	AME	OR	CI 95 %	AME	OR	CI 95 %	AME
Age	FP(1)	1.04***	(1.02–1.06)	[0.004]	1.04***	(1.02–1.06)	[0.004]	1.04***	(1.02–1.06)	[0.004]	1.04***	(1.02–1.06)	[0.004]	1.04***	(1.02–1.06)	[0.004]	1.04***	(1.02–1.06)	[0.004]
	FP(2)	0.98***	(0.97–0.99)	[−0.002]	0.98***	(0.98–0.99)	[−0.002]	0.98***	(0.97–0.99)	[−0.002]	0.98***	(0.98–0.99)	[−0.002]	0.98***	(0.98–0.99)	[−0.002]	0.98***	(0.98–0.99)	[−0.002]
Sex	Female (ref.)	–			–			–			–			–			–		
	Male	1.04	(0.92–1.16)	[0.004]	1.00	(0.89–1.12)	[0.000]	1.05	(0.93–1.17)	[0.005]	1.01	(0.90–1.13)	[0.001]	0.96	(0.85–1.08)	[0.004]	0.95	(0.85–1.07)	[−0.005]
Retirement age	Before 55				–						–			–			–		
	55–59	1.18*	(1.01–1.38)	[0.017]	1.15	(0.98–1.35)	[0.015]	1.20*	(1.02–1.40)	[0.018]	1.17	(0.99–1.36)	[0.016]	1.09	(0.92–1.28)	[0.009]	1.08	(0.92–1.27)	[0.008]
	60–64	1.28**	(1.09–1.51)	[0.027]	1.22*	(1.04–1.43)	[0.021]	1.31***	(1.12–1.54)	[0.029]	1.24**	(1.05–1.44)	[0.023]	1.12	(0.95–1.32)	[0.012]	1.11	(0.95–1.31)	[0.011]
	65 or older	1.31*	(1.05–1.63)	[0.029]	1.23	(0.99–1.53)	[0.022]	1.35**	(1.08–1.68)	[0.032]	1.26*	(1.00–1.55)	[0.025]	1.12	(0.90–1.40)	[0.012]	1.12	(0.89–1.40)	[0.012]
ADL-limitation	Not limited (ref.)				–						–			–			–		
	Limited				0.77*	(0.60–0.99)	[−0.028]				0.76**	(0.59–0.98)	[−0.029]	0.77*	(0.60–0.99)	[−0.028]	0.77*	(0.59–0.99)	[−0.028]
Mobility limitation	Not limited (ref.)				–						–			–			–		
	Limited				0.62***	(0.55–0.78)	[−0.051]				0.62***	(0.53–0.74)	[−0.051]	0.66***	(0.55–0.79)	[−0.044]	0.67***	(0.56–0.80)	[−0.043]
Occupational position	Very low (ref.)													–			–		
	Low													1.33**	(1.11–1.58)	[0.027]	1.30**	(1.09–1.55)	[0.025]
	High													1.81***	(1.44–2.28)	[0.062]	1.77***	(1.41–2.23)	[0.060]
	Very high													1.98***	(1.61–2.28)	[0.074]	1.93***	(1.56–2.37)	[0.070]
Wealth	Low (ref.)													–			–		
	Medium													1.15	(0.99–1.33)	[0.014]	1.14	(0.98–1.31)	[0.013]
	High													1.33***	(1.15–1.54)	[0.030]	1.31***	(1.13–1.51)	[0.028]
Control at work	Low(ref.)	–			–												–		
	High	1.36***	(1.13–1.64)	[0.031]	1.32**	(1.10–1.59)	[0.028]										1.12	(0.93–1.35)	[0.012]
Reward at work	Low (ref.)							–			–						–		
	High							1.47***	(1.22–1.77)	[0.038]	1.43***	(1.19–1.72)	[0.035]				1.34**	(1.11–1.62)	[0.029]

Estimates for age are based on fractional polynomials (FP, two-degree). All models are additionally adjusted for country affiliation (country dummies)

* *p* < 0.05; ** *p* < 0.01; *** *p* < 0.001

**Fig. 2:**
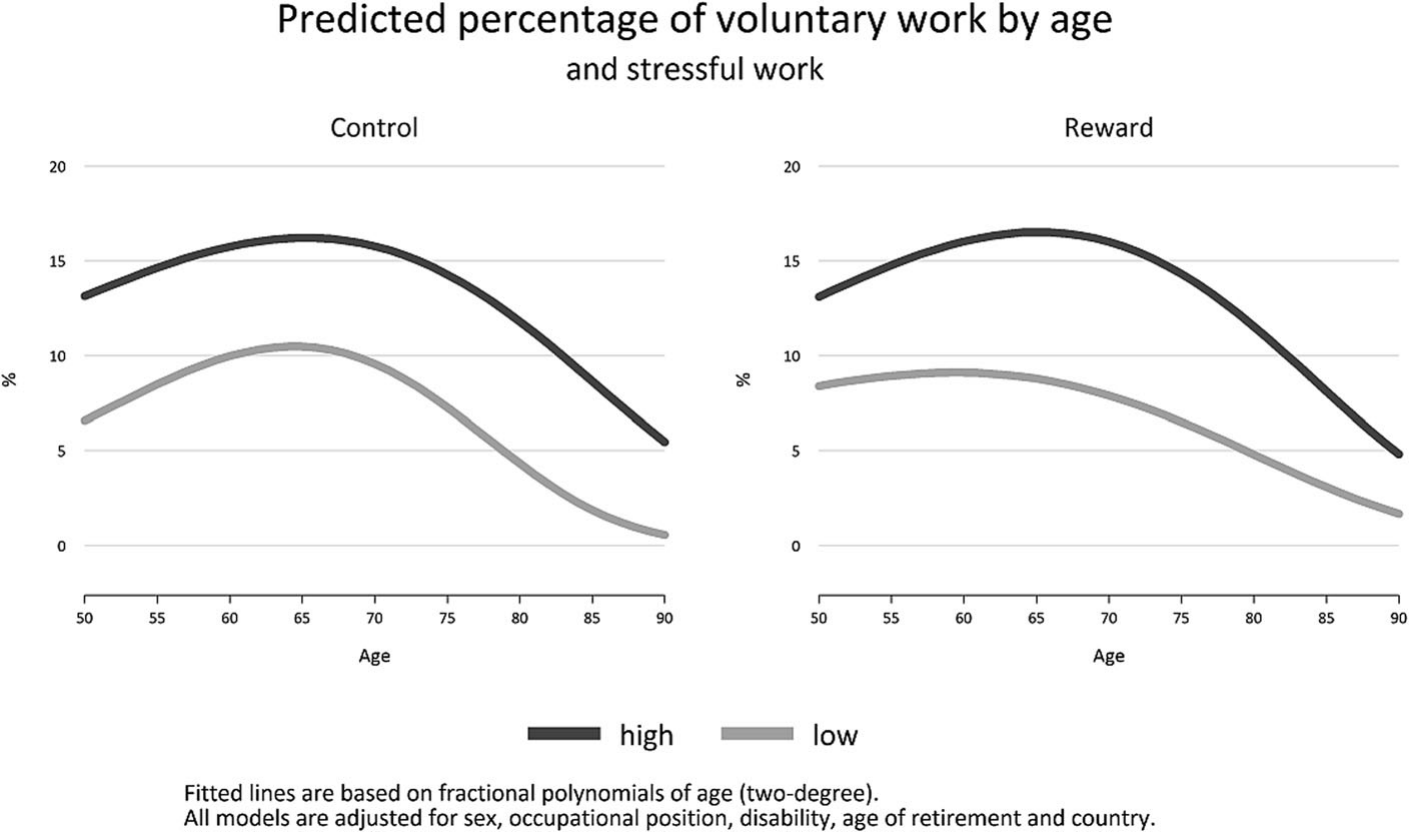
Predicted prevalence of voluntary work by age and stressful work

Finally, to summarize our main findings, we estimate the age function for each level of work stress separately and display resulting curves in Fig. 2 (Mitchell 2012), in terms of average probability of volunteering for each age (average adjusted prediction) (Williams 2012). All calculations and figures are done with STATA 13.

## Results

### Descriptive Findings

As shown in Table 1, the mean age of the sample is 68 years and it includes slightly more retired women than men (5981 vs. 5770). A third of the respondent retired before 55, and only 12 % at age 65 or older. Levels of disability were generally low, with somewhat higher levels of mobility limitations as compared to activity limitations (25 vs. 10 %). About 16 % of respondents report that they had low control or low reward during working life, and the overall percentage of older people engaging in voluntary work is 14 % in the total sample.

Turning to Table 2, we see that levels of volunteering are slightly higher levels for men, those who retired between 60 and 64, and people who have no disability limitations. Further, we see a clear social gradient of volunteering, where those with disadvantaged occupations (lower skill-levels) or lower wealth are less likely to engage in voluntary work during retirement. Finally, people who had higher levels of work stress (either low control or low reward) are less likely to participate in voluntary work after labour market exit as compared to those with low levels of work stress.

With regard to country variations, Fig. 1 shows that people are less likely to engage in voluntary work in Southern and Eastern European countries, while percentages of people volunteering are comparatively high in Northern Europe and the Netherlands. Furthermore, we see that lower participation rates in a country are generally accompanied by higher overall levels of work stress, either in terms of low control or low reward. To test this association at the individual level, we now turn to the multivariate findings.

### Multivariable Findings

Results of the logistic regression models are presented in Table 3. Taken together, four findings are worth noting: First, the results show that the association between stressful work and volunteering during retirement remains stable and significant, even after adjusting for disability in older ages. More specifically, for people who have had higher levels of control at work the odds of participating in voluntary work is 1.36 times greater than the odds for people with low control at work. In terms of average marginal effects, this is a difference of 3.1 percentage points between the two groups (AME = 0.031). Similarly, for those who had high reward in their job, percentage point differences are 3.8 (model 4). This strongly supports the assumption that people who had advantaged working conditions during working life are more likely to participate in voluntary work after labour market exit – even if disability is considered.

Second, the results reveal a clear social gradient of volunteering, where those with advantaged socioeconomic circumstances are more likely to participate in voluntary work. This is particularly true in the case of the occupational position, where the predicted levels of engagement are 7.4 percentage points higher for people in a very high position as compared to workers with a very low position (again after controlling for disability).

Third, in the final model of Table 3 (where all factors are included simultaneously) odds ratios of control become non-significant, as well as AME attenuate substantially (from 0.028 to 0.012). This indicates that our two indicators of socioeconomic circumstances and control do not affect volunteering independently, but – for example – that the effect of high control is partly due to higher wealth in older ages. In contrast, the association between high reward and volunteering remains significant - even after adjusting for both measures of socioeconomic position. Notably, the same findings are found if the final model is estimated for the two indicators of stressful work separately (results not shown) – thus – minimizing the possibility that high control becomes insignificant because of including rewards at work.

The fourth and final observation worth noting is that the two FPs of age are strongly significant in all models, clearly indicating a non-linear association between age and volunteering. This latter aspect becomes obvious in Fig. 2, where we predict the age-function for each subgroup of stressful work.

For both indicators of work stress and subgroups, we see that there is an inverted U-shaped association between age and volunteering, with highest levels of engagement between age 65 and 70. In addition, at all ages participation rates are higher among those who had good working conditions during working life as compared to those with poor conditions. In the case of low control differences are at about 5 % across all ages, while differences in participation rates appear slightly more pronounced in the case of low reward, in particular between age 60 and 75.

## Discussion

In this paper, we used data from the SHARE study and investigated associations between psychosocial work stress in midlife and participation in voluntary work during retirement. Our main assumptions were that older men and women who had good working conditions in their previous working life – as defined by two complementary indicators: high control and high reward - are also more likely to engage in productive activities during retirement (hypothesis 1), regardless of levels of disability or socioeconomic circumstances (hypothesis 2). The SHARE study provides a unique opportunity of studying these questions, with information on psychosocial work stress during working life and on participation in voluntary work among more than 11,000 retired men and women.

Our main findings can be summarized as follows: First, people who experienced stressful work in their main occupation during working life were also less likely to participate in voluntary work after labour market exit, specifically men and women who had jobs defined in terms of low control and low reward at work. This supports our first assumption and means that psychosocial stress at work is clearly linked to the likelihood of participating in productive activities after labour market exit. Our second main finding was that effects of control and reward on the likelihood to engage in voluntary work were not confounded by disability in older ages. In case of levels of work-related rewards this was also true for socioeconomic circumstances. This is important, because it shows that even after considering the impact of two further determinants of volunteering recurrent favourable experience of recognition and reward at work during midlife may motivate people to engage in productive activities at older ages. Furthermore, the finding indicates that our measure of control is more likely to be confounded by respondents’ socioeconomic position, as compared to reward. Thus, while we found clear support for both hypotheses in the case of reward, the second hypothesis was only partly supported in the case of control, where associations became non-significant once we adjusted for socioeconomic position. Two additional findings must be noted: Our results showed that people who worked in higher-skilled occupations or had higher levels of wealth were more likely to engage in voluntary work, thus revealing a social gradient in the prevalence of voluntary work. Finally, by using a flexible modulation of the age-function, we demonstrated that the association between age and volunteering is inversely U-shaped with highest levels between 65 and 70 years. In addition, we showed that higher participation rates among people with good conditions at work are observed at all stages of the life course, in particular in the case of reward between age 60 and 75.

Overall, these findings confirm existing research, specifically work which considered socio-demographic and socioeconomic characteristics in conjunction with engagement in voluntary work in older ages (Loh and Kendig 2013; Siegrist and Wahrendorf 2009; Hank and Stuck 2008; Erlinghagen and Hank 2006; McMunn et al. 2009). Yet, by linking two distinct aspects of work-related stress in midlife to volunteering during retirement, our results add both to existing literature of occupational health and to the literature of health and well-being in older ages.

To our knowledge, this is - at the conceptual level - the first study that explicitly refers to core notions of two theoretical models of work stress, which are instrumental for a positive self-regulation, and that assumes that these notions are related to the individual motivation of engaging in voluntary work during retirement. Volunteering is not the only outcome reflecting personal characteristics of retired women and men that was linked to psychosocial working conditions. In a large French cohort study, for example, components of either work stress models predicted fatigue and health functioning 8 years later when participants were retired (Wahrendorf et al. 2012; Sembajwe et al. 2012). Or, a further analysis conducted on the basis of SHARE data showed that men and women who experienced low control and low reward at work during midlife had elevated risks of depressive symptoms (Wahrendorf et al. 2013). These findings support the notion of long-term impact of adversities in core social roles in midlife on emotional, motivational and behavioral outcomes after labour market exit.

Similarly, with the current contribution we extended the time frame of existing studies investigating determinants of volunteering in older ages by additionally considering earlier stages of the life course, specifically work-related factors during midlife. This supplements research on health in older ages (Mc Munn et al. 2006) by highlighting that opportunities and capabilities to achieve an active and healthy ageing are also linked to conditions at earlier stages of the life course (Wahrendorf et al. 2013; Platts et al. 2013; Blane et al. 2012; Blane 2006). Nevertheless, we do not pretend that productive activities such as volunteering are the only way of successfully coping with the ageing process, in particular as many older are excluded from this option.

Although our study profits from several strengths (theoretical approach, study design and adjustments for important confounders), we have to consider several limitations. First, while this contribution focussed on proximal determinants of volunteering, our results also point to existing variations of volunteering and of work stress between countries. This raises the question how distal determinants, such as national policy regulations, may explain these country-differences, with at least three explanations discussed in the literature (Hank 2011; Salamon and Sokolowski 2003), and a possible fourth based on our findings: First, it may be that higher levels of engagement are due to higher levels of social expenditures in a country, because expenditures and related programs are related to better health and personal resources, which in turn increase the likelihood of an engagement. Or, a second explanation could be that social expenditures and related programs create an infrastructure that allow individuals to engage in voluntary work, because the state takes care of specific responsibilities (e.g. providing care for family members), and thus provide more opportunities for individuals. Thirdly, it may be that the voluntary sectors itself including its cultural foundation do very between countries. Fourth, and against the background of our study, labour market policies may be important. In recent studies these types of policies were related to favourable working conditions, in particular active labour market policies that promote further education and invest into supported employment and rehabilitation (Lunau et al. 2013; Wahrendorf and Siegrist 2014). Thus, by promoting favourable working conditions, labour market policies may indirectly be related to levels of volunteering as well.

A second limitation refers to additional variables that we may have included in our analyses, for example engagements in voluntary work at earlier stages of the life course, and in particular at the beginning of the retirement transition period. In that respect, there is evidence that prior engagement is an important predictor of volunteering during retirement (Erlinghagen 2010), and furthermore that people are more likely to combine paid work and volunteering at later stages of their working life, if they are enabled to remain working (probably under good conditions) with reduced working hours (Sugihara et al. 2008). Yet, information on voluntary work during working life was not available in the SHARELIFE data, and thus, we could not address this aspect. Future analyses based on upcoming waves of SHARE may address this aspect in more detail, in particular by studying individuals’ histories of volunteering in older ages.

Third, survey participation was not very high in some countries and a selection bias may have affected our results to some extent. Yet, response rates were above average and analyses comparing the SHARE sample to other prominent European surveys (e.g. the European Social Survey) confirmed that the sample represents the general population quite well (Börsch-Supan and Mariuzzo 2005). Furthermore, while it is possible that a selection bias lead to higher levels of volunteering (because volunteers are also more likely to participate in surveys), it is unlikely that a selection bias affected the associations between work stress and volunteering.

Fourth, some core measures of our study were collected retrospectively, in particular stressful work and occupational position during working life. As a consequence, we need to consider a potential recall bias, where information may be positively tuned or not remembered accurately. Yet, a high prevalence of lower occupational positions and levels of work stress comparable to other European studies (Niedhammer et al. 2012) do not support this argument, as well as numerous studies have shown that retrospective data (in particular those collected via “lifegrid” as in SHARELIFE) provides reliable and valid information (e.g.: Havari and Mazzona 2011; Berney and Blane 1997; Belli et al. 2007). Nevertheless, additional data allowing for bias control due to distinct personality characteristics would have been desirable, but was not available in this study.

Finally, we have to ask if our results can be transferred to other generations and other countries. In fact, although we covered a range of European countries with different political and cultural histories, our sample was restricted to older European that grew up under specific circumstances (e.g. second world war). Therefore, future studies may test if our results can be replicated for other countries and for other generations.

In conclusion, this study demonstrates that engagement in voluntary work is related to psychosocial working conditions during midlife, in particular low reward and low control at work. This suggests that promoting working conditions may not only increase health and well-being, but also encourage participation in productive activities after labour market exit.

## Electronic supplementary material

ESM 1 (DOC 28 kb)
