# Supplementary material for: Linking Quality of Work in Midlife to Volunteering During Retirement: a European Study
Source: J Popul Ageing. 2015 Jul 8;9:113–30. doi: 10.1007/s12062-015-9129-8 (PMC4785201; doi:10.1007/s12062-015-9129-8)
Supplement: Supplementary file 1 — (DOC 28 kb) [file 12062_2015_9129_MOESM1_ESM.doc]

Table S1. Measures of stressful work during adulthood

| **Dimension** | **Item** |  |
| --- | --- | --- |
| Control | - I had very little freedom to decide how to do my work. - I had an opportunity to develop new skills. |  |
| Reward | - I received the recognition I deserved for my work. - Considering all my efforts and achievements, my salary  was adequate. |  |
